# S10-3: Promoting biking in secondary schools – evaluating the bikepool project

**DOI:** 10.1093/eurpub/ckae114.243

**Published:** 2024-09-26

**Authors:** Karim Abu-Omar, Tobias Fleuren, Maike Till, Raluca Sommer, Heiko Ziemainz

**Affiliations:** Department of Sport Science and Sport, Friedrich-Alexander-Universität Erlangen-Nürnberg, Germany; Department of Sport Science and Sport, Friedrich-Alexander-Universität Erlangen-Nürnberg, Germany; Department of Sport Science and Sport, Friedrich-Alexander-Universität Erlangen-Nürnberg, Germany; Department of Sport Science and Sport, Friedrich-Alexander-Universität Erlangen-Nürnberg, Germany; Department of Sport Science and Sport, Friedrich-Alexander-Universität Erlangen-Nürnberg, Germany

## Abstract

**Purpose:**

Teaching children and adolescents how to bike safely in traffic is a key skill that supports active mobility and contributes to decarbonizing the transport sector. Within the paradigm of transformative science, it is important to focus efforts on supporting practice-based projects that operate well in real-live, but lack scientific evaluation of their potential effectiveness.

Bikepool, a project that evolved out of practice, is run by a volunteer organization and has been scaled to over 166 schools in the past 12 years. Despite its success, there has been no assessment of its potential effects on pupils, teachers, and the school environment. This presentation highlights an evaluation study set to begin in mid-2024.

**Methods:**

The planned evaluation study uses a mixed method approach and builds on the methodology of co-production and participation. Qualitative interviews with teachers will be conducted in 15 schools. Pupils will take part in designing the evaluation. A quantitative survey will assess the potential effects of bikepool in all schools.

**Results:**

Bikepool provides schools with bikes, safety equipment, a bike repair shop, and vocational training for teachers. The project’s evaluation, grounded in transformative science, follows three phases: (1) understanding the project and mutual learning between researchers and project staff, (2) co-creating an evaluation plan with project staff and pupils, and (3) collecting and analyzing data. Researchers will support the project in optimizing its work and identifying ways to scale the project to additional schools.

**Conclusion:**

Practice-based projects demonstrating real-live applicability and a high reach offer an excellent opportunity for transformative science. Supporting such projects to assess and optimize their effectiveness and help them scale can increase their public health impact. At the same time, the paradigm of transformative science is still evolving and practical experience on how to make use of it is still lacking.

**Funding:**

The project is funded by Verein der Freunde und Förderer des Bikepool Hessen e.V.

